# Association of Phoenixin-14 with Body Mass Index Categories and Proteinuria in Adults with Type 2 Diabetes

**DOI:** 10.3390/medicina62040697

**Published:** 2026-04-04

**Authors:** Esra Suay Timurkaan, Hakan Ayyıldız, Mehmet Buğra Bozan, Muhammed Fuad Uslu, Mustafa Timurkaan

**Affiliations:** 1 Department of Internal Medicine, Fethi Sekin City Hospital, 23280 Elazig, Turkey; dresrasuay@gmail.com (E.S.T.); dr.fuslu@gmail.com (M.F.U.); 2 Department of Biochemistry, Fethi Sekin City Hospital, 23280 Elazig, Turkey; hknayyildiz@hotmail.com; 3 Department of General Surgery, Fethi Sekin City Hospital, 23280 Elazig, Turkey; bbozan@yahoo.com

**Keywords:** phoenixin-14, type 2 diabetes mellitus, obesity, body mass index, proteinuria

## Abstract

*Background and Objectives*: Human data on obesity and type 2 diabetes mellitus (T2DM) remain limited and inconsistent, particularly with respect to adiposity-related phenotypes and renal involvement. We aimed to assess PNX-14 across body mass index (BMI) categories and to investigate its associations with BMI, insulin resistance indicators, and proteinuria in adults with T2DM. *Materials and Methods*: In this prospective cross-sectional study, participants were classified into four groups according to World Health Organization body mass index (BMI) thresholds: 18.5–24.9, 25.0–29.9, 30.0–34.9, and ≥35.0 kg/m^2^. Serum PNX-14 was measured using a human ELISA kit. Group comparisons, trend analysis, false discovery rate-adjusted Spearman correlations, HC3-robust multivariable regression, and parsimonious structural equation modeling were performed. *Results*: PNX-14 differed significantly across BMI categories and increased monotonically with increasing BMI (*p* < 0.001 for both the overall comparison and the trend). PNX-14 showed a positive correlation with BMI (ρ = 0.491; qFDR < 0.001), whereas no significant relationship was observed with insulin or HOMA-IR after FDR correction (qFDR = 0.795 for insulin and qFDR = 0.793 for HOMA-IR). In the model adjusted for age, sex, and BMI, higher PNX-14 was independently associated with lower proteinuria (β = −0.326, 95% CI −0.584 to −0.067; *p* < 0.05), whereas BMI was positively associated with proteinuria (β = 0.407, 95% CI 0.132 to 0.682; *p* < 0.01). Structural equation modeling supported positive BMI→PNX-14 and BMI → proteinuria paths, a negative PNX-14→proteinuria path, and a non-significant PNX-14→HOMA-IR path. *Conclusions*: In adults with T2DM, PNX-14 appears to be more consistently related to adiposity than to glycemic or insulin resistance indicators. However, when evaluated together with proteinuria, it may offer a testable framework for phenotyping based on renal involvement within the obesity spectrum. Nevertheless, this approach needs to be validated in studies from different centers and with repeated measurements.

## 1. Introduction

Obesity is a multifactorial, chronic disease that is rapidly increasing in modern societies and is associated with substantial morbidity and mortality through metabolic syndrome, type 2 diabetes, and cardiovascular disease [1,2]. The global burden of obesity and its related comorbidities has serious consequences for both individuals and health systems [3]. Insulin resistance, chronic inflammation, and modified neuroendocrine signaling are pivotal in its pathogenesis [4,5]. Current information on overweight and obesity levels and trends remains important not only for measuring health effects but also for encouraging decision-makers to act and for identifying areas in which progress has, or has not, been achieved [6].

Phoenixin-14 (PNX) is a neuropeptide first identified in 2013 [7]. Phoenixin-14 has been documented to be expressed in various brain nuclei, especially the hypothalamus, in addition to peripheral tissues like the small intestine and pancreas. Metabolically, it has been associated with various physiological processes, including energy homeostasis, appetite regulation, reproductive function, stress response, and inflammatory mechanisms [8,9,10]. PNX administration, studied mainly in animal models, has been shown to increase food intake and to influence energy balance through orexigenic neurons. Human data, however, remain limited and largely heterogeneous. A few preclinical studies have provided indirect findings linking phoenixin to the autonomic nervous system, thereby laying the groundwork for human studies and suggesting associations with appetite and central autonomic regulation. It has also been shown that phoenixin supports white adipogenesis and may therefore contribute to the regulation of body mass [11,12]. These findings have drawn particular attention to the possible role of PNX in metabolic processes.

There is not yet any data from studies in which the clear role of PNX in metabolic processes has been examined compared with other neuropeptides. For this reason, its effects are still controversial, and further human studies are needed. In human studies conducted so far, it has been reported that serum phoenixin-14 levels were increased in women with obesity and polycystic ovary syndrome and that this increase showed a significant positive correlation with parameters such as BMI, HOMA-IR, LH, and testosterone [13,14]. This suggests that phoenixin may be associated with insulin resistance and metabolic dysfunction. In addition, a comprehensive review study revealed that phoenixin may assume a broader role in metabolic processes such as energy homeostasis and glucose and lipid metabolism in humans, indicating its potential as a biomarker for assessing metabolic health and disease risk associated with obesity. It is considered in the literature that there is a need for biomarkers that can show metabolic risk and disease progression earlier and more accurately in terms of diseases secondary to obesity [15,16].

The primary objective of this study was to compare PNX-14 levels across different BMI categories in adults with T2DM. The secondary objectives included assessing correlations with metabolic markers and examining the association between PNX-14 and proteinuria, adjusted for BMI.

## 2. Materials and Methods

### 2.1. Study Design and Ethical Approval

This study was designed as a prospective cross-sectional study. It was conducted in collaboration between the Department of Internal Medicine and the Biochemistry Laboratory at Elazığ Fethi Sekin City Hospital. Following the approval of the local ethics committee (Date: 6 June 2024; Decision No: 2024/09-25), patients with T2DM who presented consecutively to the Internal Medicine Outpatient Clinic between 1 July 2024 and 1 January 2025 were evaluated. All clinical and laboratory measurements were collected at a single time point during the admission visit. Written informed consent was obtained from all participants.

### 2.2. Selection of Participants

*Inclusion Criteria:* The inclusion criteria included age 18–65 years, BMI > 18.5 kg/m^2^, and a diagnosis of type 2 diabetes treated with oral antidiabetic agents. Patients receiving renin–angiotensin–aldosterone system (RAAS) blocking agents were not included in order to minimize treatment-related confounding on urinary protein excretion. Patients receiving sodium–glucose cotransporter-2 (SGLT2) inhibitors were also excluded to reduce potential treatment-related effects on glucosuria and urinary protein excretion. The study population consisted of adults with a previously established clinical diagnosis of T2DM. Accordingly, individuals with a known diagnosis of T1DM or with clinical suspicion of monogenic diabetes were not included in the study cohort.

*Exclusion Criteria:* The exclusion criteria included pregnancy, lactation, chronic liver failure, cancer, autoimmune disease, and endocrinopathies.

### 2.3. Clinical and Demographic Data Collection

Age, sex, medical history, smoking, and alcohol habits were recorded. Alcohol consumption was recorded as part of the clinical history. However, it was not further subclassified in the absence of abnormal liver function tests or other exclusionary clinical conditions. Height and body weight were measured with calibrated devices; BMI (kg/m^2^) was calculated. Blood pressure was measured in the morning after a brief seated rest using a calibrated automated device under routine outpatient clinic conditions.

### 2.4. BMI Classification

BMI categories were defined according to the World Health Organization classification thresholds [17]. Participants were categorized into four groups: Group 1, normal weight (BMI 18.5–24.9 kg/m^2^; n = 23); Group 2, overweight (BMI 25.0–29.9 kg/m^2^; n = 36); Group 3, obese (BMI 30.0–34.9 kg/m^2^; n = 35); and Group 4, morbid obesity (BMI ≥ 35.0 kg/m^2^; n = 14).

### 2.5. Laboratory Procedures

#### 2.5.1. Blood Sample Collection and Processing

Fasting blood samples (8–12 h) were collected from all study participants and placed in tubes containing aprotinin (BD Vacutainer SST II Advance, BD, Plymouth, UK). After collection, samples were centrifuged at 4000 rpm for 10 min. Serum aliquots were then transferred into Eppendorf tubes and stored at −20 °C until PNX-14 quantification, with each sample thawed only once.

#### 2.5.2. Biochemical Analyses and Calculations

Serum glucose, AST, ALT, urea, creatinine, total cholesterol, HDL cholesterol, LDL cholesterol, triglycerides, and urinary total protein and creatinine were measured by spectrophotometric methods using an AU5800 analyzer (Beckman Coulter, Inc., Miami, FL, USA). Insulin and C-peptide were measured by chemiluminescent immunoassay on the DxI 800 system (Beckman Coulter, Inc., Miami, FL, USA), while HbA1c was determined by capillary zone electrophoresis (Sebia Capillarys 3 Tera, Lisses, France). Urinary total protein and creatinine were measured by standard automated methods. In this study, urinary protein assessment was based on the spot urine protein-to-creatinine ratio obtained from a single sample collected at the same visit. HOMA-IR was calculated using the formula [fasting glucose (mg/dL) × fasting insulin (μIU/mL)]/405. BMI was calculated manually by dividing body weight (kg) by height squared (m^2^) and was then entered into the system. Serum phoenixin-14 (PNX, pg/mL) levels were measured in duplicate, in a blinded manner, and in a single analytical run using a validated commercial ELISA kit (BT Lab, Bioassay Technology Laboratory; Catalog No: E7050Hu, Shanghai, China), in accordance with the manufacturer’s protocol. Absorbance measurements were performed with a Chromate 4300 microplate reader (Awareness Technology, Palm City, FL, USA).

### 2.6. Data Management and Power Analysis

The data were anonymized and stored securely. For the primary endpoint, defined as detecting a 20% difference among four groups (α = 0.05 and β = 0.20 [80% power]), the study was initiated with a target sample of 130 patients to detect a medium-to-large effect size (Cohen’s f ≈ 0.30). After excluding 14 patients who withdrew voluntarily and 8 patients who were excluded because of sample-related or technical problems during biochemical analysis, the study was completed with the remaining 108 patients. All procedures were carried out in accordance with institutional and national ethical standards, the 1964 Helsinki Declaration and its later amendments, and the STROBE guideline for observational studies [18].

### 2.7. Statistical Analysis

All analyses were conducted in R within the RStudio version 4.5.1. environment. The Shapiro–Wilk test and visual assessments were utilized to evaluate the distributional assumptions for continuous variables. Continuous variables are presented as mean (SD) for descriptive readability, whereas between-group comparisons were performed using non-parametric methods when distributional assumptions were not met. Categorical variables are presented as n (%). Kruskal–Wallis tests were used to compare body mass index (BMI) categories for continuous variables. The Jonckheere–Terpstra test was applied to examine ordered trends among BMI groups. Effect sizes were reported whenever feasible. Spearman correlation coefficients were computed to analyze the relationships between PNX-14 and various clinical or metabolic characteristics. An adjustment for the false discovery rate (FDR) was incorporated. We employed standardized estimators (for each 1 SD increase) and HC3 robust standard errors to construct multivariable linear regression models. Structural equation modeling (SEM) was employed to investigate theoretical relationships among BMI, PNX-14, and renal or metabolic outcomes. The SEM analysis was performed in R (version 4.5.1) using the lavaan package (version 0.6-16). To examine indirect effects, bootstrap resampling with 1000 iterations was utilized in the SEM analysis. The final SEM model had a saturated structure (degrees of freedom = 0). All tests were two-tailed, and the threshold for statistical significance was set at *p* < 0.05.

## 3. Results

### 3.1. Study Population and Baseline Clinical Characteristics

A total of 108 participants were included in the study. The participants were classified according to BMI groups as Group 1 (normal weight, n = 23), Group 2 (overweight, n = 36), Group 3 (obese, n = 35), and Group 4 (morbidly obese, n = 14). Age and sex distributions did not differ significantly across BMI groups. Demographic and baseline clinical characteristics according to BMI groups are presented in Table 1.

### 3.2. PNX Levels According to BMI Groups

**Figure 1:**
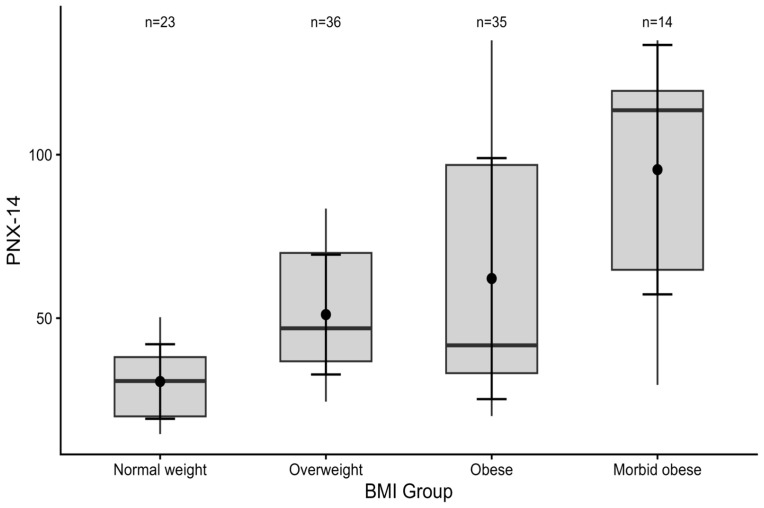
Distribution of PNX according to BMI groups. The boxplots show the distribution of PNX-14 across BMI categories. Boxes represent the interquartile range, center lines indicate medians, and numbers above the boxes denote group sample sizes.

PNX levels showed a significant difference among BMI groups (Kruskal–Wallis *p* < 0.001). A monotonic increasing trend in PNX levels was detected as BMI increased. Ordered trend analysis showed that this increase was directional and consistent (Jonckheere–Terpstra *p* < 0.001, ε^2^ = 0.267). These findings reveal that the relationship between BMI and PNX levels is not limited only to group differences but exhibits a continuing trend across obesity severity (Figure 1).

### 3.3. Relationships Between PNX and Metabolic Markers

**Figure 2:**
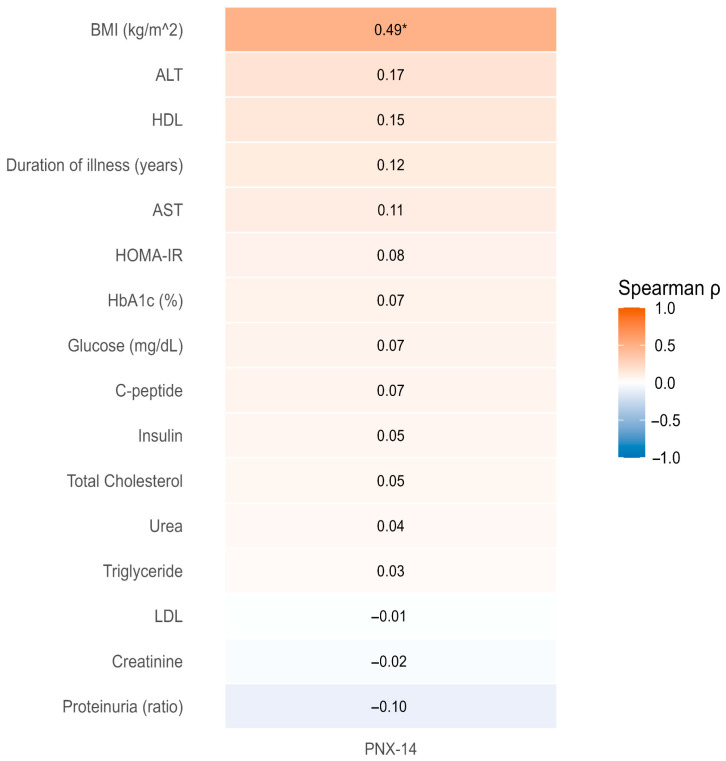
PNX correlation heatmap. The heatmap displays Spearman correlation coefficients (ρ). FDR correction was applied for multiple comparisons. The color scale reflects the direction and magnitude of the correlations (PNX-14: pg/mL). * ρ = 0.491; qFDR < 0.001.

The relationships between PNX-14 levels and clinical/biochemical variables were evaluated by Spearman correlation analysis, and FDR correction was applied for multiple comparisons. A positive correlation was detected between PNX-14 and BMI (ρ = 0.491; qFDR < 0.001). In contrast, no significant relationship was observed for PNX-14 with insulin and HOMA-IR after FDR correction (qFDR = 0.795 for insulin and qFDR = 0.793 for HOMA-IR). The relationships with other biochemical parameters were generally weak and did not show significance after multiple comparison correction. The overall correlation profile is summarized in Figure 2.

### 3.4. Relationship Between PNX and Proteinuria

The relationship between PNX and proteinuria was evaluated with multivariable regression models using robust standard errors. In Model 1, no significant relationship was detected between PNX (per 1 SD increase) and proteinuria; age was positively associated with proteinuria. In Model 2, in which BMI was added, PNX became inversely and independently associated with proteinuria; the significance of age was preserved.

In the model adjusted for age and sex, the association was weak. After additional adjustment for BMI, higher PNX (per 1 SD increase) was independently associated with lower proteinuria (β = −0.326; 95% CI −0.584 to −0.067; *p* < 0.05). This relationship continued after controlling for potential confounders. In the same model, BMI (per 1 SD increase) was positively associated with proteinuria (β = 0.407; 95% CI 0.132 to 0.682; *p* < 0.01). Detailed model estimates are presented in Table 2.

### 3.5. Relationships Between Variables by Structural Equation Modeling

**Figure 3:**
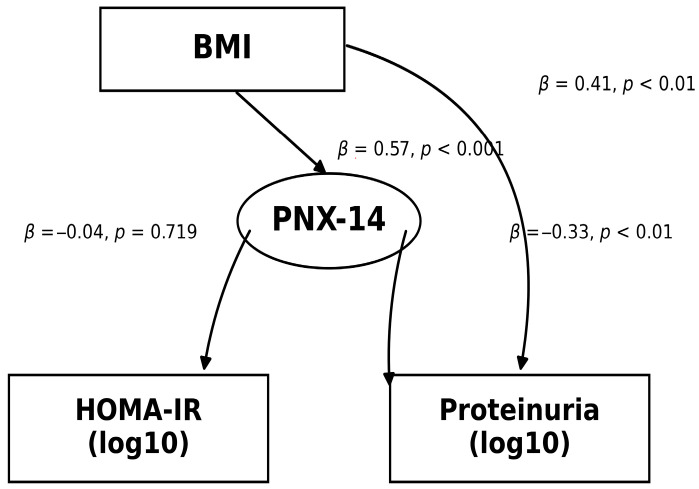
Parsimonious structural equation model. The model includes the BMI→PNX-14, PNX-14→proteinuria (log10_prot_z), and BMI→proteinuria (log10_prot_z) paths; it also includes the PNX-14→HOMA-IR (log10_HOMA-IR (z)) path. The arrows show the directional relationships tested in the model. The values next to the arrows are standardized path coefficients (Std β). The relevant *p*-values are given next to the arrow for each path.

The relationships among PNX, BMI, and proteinuria were evaluated using structural equation modeling. Accordingly, in the parsimonious SEM, a positive relationship was observed between BMI_z and PNX_z (Std β = 0.571; *p* < 0.001). PNX_z was inversely related to proteinuria (log10_prot_z) (Std β = −0.326; *p* < 0.01), and the direct effect of BMI_z on proteinuria was positive (Std β = 0.407; *p* < 0.01). The path between PNX_z and HOMA-IR (log10_HOMA-IR (z)) was not significant (Std β = −0.039; *p* = 0.719). The model diagram is presented in Figure 3. Indirect effect analysis indicated that BMI had a negative and significant indirect effect on proteinuria through PNX (ind_prot: Std β = β = −0.186; *p* < 0.05). The indirect effect of BMI on HOMA-IR through PNX was not significant (ind_HOMAIR: Std β = −0.022; *p* = 0.732).

## 4. Discussion

Our findings suggest a framework in which PNX is more consistently aligned with the degree of adiposity in the clinical setting, whereas it does not provide an equally clear discriminative signal along the glycemic/insulin resistance axis. This finding has an important implication. Although PNX is still being evaluated in an area where human data are limited and heterogeneous, it may reflect multidimensional relationships within the metabolic setting. The variability of the association between PNX and glycemic parameters across different populations suggests that PNX may need to be interpreted more in conjunction with phenotypic components than with metabolic dysfunction alone. Taken together, this pattern suggests that PNX, rather than behaving like a classical glycoregulatory peptide that mirrors metabolic stress markers, may function primarily as a biomarker sensitive to the adiposity-related phenotype.

The potential role of PNX in neuroinflammatory responses, PCOS, insulin resistance, and cardiovascular diseases has been addressed in several studies [14,15,19]. Ullah and colleagues reported that serum phoenixin-14 levels in women with polycystic ovary syndrome were higher than those in control groups and were positively correlated with LH and nesfatin-1 [14]. Friedrich and colleagues showed that PNX may be regulated by inflammatory signals and stress-induced neuroinflammation [19]. In another study, it was suggested that proinflammatory cytokines, stress-related factors, and other neuroimmune signals may regulate phoenixin (PNX) gene expression in the central nervous system [20]. Our finding of a positive correlation between PNX and BMI is in line with earlier evidence suggesting that PNX participates in neuropeptidergic regulation during adipose tissue expansion and in appetite and energy storage [12,19]. Although animal models have shown that hypothalamic PNX expression may change in response to metabolic signals such as food intake and fatty acid levels, the literature still indicates that the effect of PNX on appetite control across species remains uncertain [20]. In another study conducted in patients with anorexia nervosa, PNX levels decreased with weight loss and increased again with partial weight regain [21]. In human studies, serum phoenixin-14 levels have mostly been evaluated in the setting of obesity and PCOS, and levels have been reported to increase in these groups and to correlate positively with BMI [13,14]. Clinical data in individuals with type 2 diabetes are more limited, and some case–control studies have reported lower PNX-14 levels together with an inverse relationship with BMI [22]. Therefore, in our study of adults with type 2 diabetes, we examine how PNX-14 varies across BMI categories and how it may be interpreted alongside proteinuria to help make the differing findings in the literature more clinically understandable. It also suggests that PNX may be related to the degree of adiposity as reflected by BMI categories. Nonetheless, we contend that it remains premature to establish a clinical decision threshold based solely on PNX, as additional research is required to ascertain its reliability and validity across various populations and clinical scenarios. The absence of a definitive parallel relationship between PNX and indicators of insulin resistance implies that certain positive correlations documented in the literature may not be applicable across all populations or clinical contexts. This divergence strengthens the view that the PNX–phenotype relationship may be sensitive to the participant profile and clinical setting. In our treated type 2 diabetes population in particular, the stronger influence of clinical and treatment-related factors on glycemic parameters and insulin measurements may have attenuated the link between PNX and metabolic impairment. These observations support the view that PNX-14 may not represent a broad biochemical signal in treated T2DM and that its clinical relevance may be more evident within the adiposity-related phenotype. At present, PNX cannot replace the clinical assessments that summarize body weight and the number and type of accompanying diseases. At most, if supported by future validation studies, it may serve as a complementary measurement that helps classify obesity phenotypes more precisely.

The most distinctive finding of this study is that the relationship between PNX and indicators of renal involvement may take on a different clinical meaning once adiposity is taken into account. Obesity-related kidney damage and the proteinuria spectrum may progress silently in clinical practice, which increases interest in new biomarker candidates that could support earlier risk discrimination in this area. For this reason, in models that interpret PNX together with renal involvement, accounting for BMI in addition to age and sex helps place the relationship in a more accurate clinical frame. Still, the central discussion is whether PNX will remain a signal reflecting obesity severity or whether it may behave as a complementary marker candidate capable of separating clinically meaningful subgroups with different degrees of renal involvement at the same level of adiposity. Our findings suggest that the second possibility is at least worth investigating. According to our results, there may be subgroups that differ in terms of renal involvement despite similar BMI levels, and PNX may carry a signal accompanying this divergence. Nevertheless, this interpretation requires caution. Given the heterogeneity of our dataset and the lack of external validation, these findings cannot support a clinical decision claim. However, as obesity-related renal involvement becomes more common, they raise clinically relevant research questions about further personalizing the intensity of screening and follow-up. The component of PNX that is independent of BMI may align differently with the renal phenotype. An important internal consistency inspection of this framework is also visible in the SEM findings: the positive BMI→PNX path, the inverse PNX→proteinuria path, the positive BMI→proteinuria path, and the non-significant PNX→HOMA-IR path all support the same structural message. In addition, the significant negative indirect effect of BMI on proteinuria through PNX further supports the idea of a discriminative signal that may be read bidirectionally on the renal axis. Even so, while the positive association between BMI and proteinuria remained in the expected direction, the reversal of the PNX–proteinuria relationship after BMI adjustment is compatible with a masking-like situation. Accordingly, these findings should be interpreted with additional caution. In contrast, the question of whether PNX measurement may help personalize the timing and intensity of renal screening in the setting of obesity remains clinically relevant. Because obesity-related glomerular damage and proteinuria are more frequent, early detection of renal involvement is clinically important [23,24].

Accordingly, the next step is to test the reproducibility of these findings across different centers and clinical spectra. It is important to re-evaluate the inverse relationship between PNX and proteinuria using renal measurements obtained in the same individuals at different time points. This will help clarify how clinically reliable the observed relationship actually is. In addition, separate analyses according to treatment subgroups and accompanying diseases may show more clearly in which clinical settings PNX may truly carry discriminative information. Likewise, adding direct body composition measurements alongside BMI may help clarify which dimensions of adiposity are more closely aligned with PNX. If these signals are validated in independent studies, it may become more reasonable to test PNX as a measurement that could help define patient subgroups or distinguish levels of risk in obesity research. However, caution is needed when generating or interpreting a threshold for clinical use from the data of the present study. The sample size may be insufficient to detect rarer or lower-prevalence relationships. In addition, because of the cross-sectional design, the observed relationships cannot be interpreted directly in terms of directionality or causality. The single measurement of proteinuria is also more vulnerable to biological and methodological variability. Similarly, the absence of repeated biochemical and renal measurements limits the ability to assess the temporal stability of these findings. Consistent with this, renal assessment in the present study was based on a single-visit spot urine protein-to-creatinine ratio, and additional renal function measures were not incorporated into the predefined analytic framework. When treatment details, lifestyle factors, and the burden of accompanying diseases are not sufficiently disentangled, the possibility that the results were influenced by other factors cannot be completely excluded. The absence of direct body composition measurements leaves subdimensions of the PNX–adiposity relationship, such as fat distribution, unobserved. Finally, although the SEM approach helps summarize the relationships within a single framework, additional checks are needed to show whether similar results are preserved across different analytical setups. These limitations may help shape the design of subsequent studies more clearly.

## 5. Conclusions

This study suggests that, in the clinical setting, it may be more reasonable to view PNX as a signal that changes most meaningfully with adiposity. However, there is not yet sufficient evidence to position PNX as a marker that summarizes the glycemic/insulin resistance axis. Therefore, at present, PNX should be interpreted primarily as a signal related to adiposity rather than as a clinical tool for monitoring body composition. The main contribution of this study to the literature is that PNX, when interpreted together with renal involvement rather than through level differences alone, may point to a new testable framework for phenotyping within the obesity spectrum. The important aspect of this approach is that it positions PNX not as a stand-alone measurement that determines clinical decisions but as a complementary candidate marker that, if validated, may help risk stratification and define patient subgroups more clearly.

## Figures and Tables

**Table 1:** Demographic and clinical characteristics according to BMI groups.

Variable	Normal Weight	Overweight	Obese	Morbidly Obese	*p* Value
Sex	Male: 12 (52.2%)	Male: 16 (44.4%)	Male: 12 (34.3%)	Male: 8 (57.1%)	0.402
Age (years)	55.52 ± 11.90	60.25 ± 13.24	56.09 ± 8.95	57.00 ± 11.03	0.347
Duration of illness (years)	6.74 ± 5.59	9.58 ± 7.22	7.69 ± 5.52	9.43 ± 4.86	0.218
BMI (kg/m^2^)	23.22 ± 1.47	27.37 ± 1.32	33.21 ± 2.71	42.79 ± 1.96	<0.001
PNX-14 (pg/mL)	30.65 ± 11.39	51.10 ± 18.30	62.11 ± 36.84	95.42 ± 38.15	<0.001
Glucose (mg/dL)	178.39 ± 85.49	144.72 ± 46.40	152.49 ± 49.83	198.57 ± 73.49	0.103
HbA1c (%)	8.48 ± 2.48	7.75 ± 1.70	7.84 ± 1.42	9.03 ± 2.23	0.196
Insulin (mIU/L)	3.68 ± 1.95	3.32 ± 1.54	4.57 ± 1.07	3.66 ± 1.34	0.128
C-peptide (ug/L)	8.81 ± 9.71	8.61 ± 6.36	11.24 ± 7.51	7.90 ± 2.44	0.083
HOMA-IR	2.48 ± 1.78	2.80 ± 1.80	2.90 ± 1.85	2.61 ± 1.31	0.374
Triglyceride (mg/dL)	175.30 ± 60.79	199.08 ± 71.03	212.66 ± 74.59	202.29 ± 85.21	0.634
HDL (mg/dL)	49.96 ± 8.58	45.17 ± 9.03	47.09 ± 9.44	50.50 ± 10.12	0.146
LDL (mg/dL)	121.17 ± 30.09	118.28 ± 28.09	106.46 ± 41.71	117.07 ± 37.45	0.363
Total cholesterol (mg/dL)	204.00 ± 35.33	196.86 ± 42.30	189.23 ± 47.63	202.64 ± 54.28	0.604
AST (U/L)	18.91 ± 4.86	24.64 ± 11.31	23.91 ± 6.59	26.29 ± 11.72	<0.05
ALT (U/L)	17.04 ± 5.69	23.27 ± 14.34	26.94 ± 10.11	27.89 ± 18.00	<0.001
Urea (mg/dL)	30.17 ± 9.97	36.69 ± 11.99	32.74 ± 12.21	35.29 ± 13.52	0.171
Creatinine (mg/dL)	0.69 ± 0.17	0.761 ± 0.24	0.749 ± 0.21	0.670 ± 0.20	0.346
Proteinuria (ratio)	0.21 ± 0.27	0.17 ± 0.21	0.21 ± 0.30	0.45 ± 0.61	<0.05

Continuous variables are presented as mean (SD), and categorical variables as n (%). For between-group comparisons, the Kruskal–Wallis test was used for continuous variables and the Pearson chi-square test was used for categorical variables.

**Table 2:** Multivariable regression analysis of the relationship between PNX and proteinuria (HC3 robust regression).

Term	Model 1: β (95% CI)	*p*	Model 2: β (95% CI)	*p*
Intercept	−0.05 (−0.298 to 0.199)	0.692	−0.062 (−0.29 to 0.167)	0.592
PNX-14	−0.094 (−0.317 to 0.13)	0.408	−0.326 (−0.584 to −0.067)	<0.05
Age	0.268 (0.08 to 0.455)	<0.01	0.303 (0.125 to 0.481)	<0.01
Male (vs. female)	0.112 (−0.254 to 0.478)	0.545	0.139 (−0.215 to 0.493)	0.438
BMI			0.407 (0.132 to 0.682)	<0.01

1. Outcome: The proteinuria variable was included in the model after log10 transformation. This regression model was specifically constructed for urinary protein assessment based on the spot urine protein-to-creatinine ratio obtained from a single sample collected at the same visit. 2. Continuous variables were standardized per 1 SD increase. 3. Model 1 included PNX-14, age, and sex; Model 2 included Model 1 plus BMI . 4. Coefficients were reported with HC3 robust standard errors (n = 108). Adjusted R^2^ values were 0.050 for Model 1 and 0.156 for Model 2.

## Data Availability

Data are subject to third-party restrictions.

## Abbreviations

The following abbreviations are used in this manuscript:

β	Standardized regression coefficient
BMI	Body Mass Index
CI	Confidence Interval
ELISA	Enzyme-Linked Immunosorbent Assay
HbA1c	Hemoglobin A1c
HDL	High-Density Lipoprotein
HOMA-IR	Homeostasis Model Assessment of Insulin Resistance
LDL	Low-Density Lipoprotein
PCOS	Polycystic Ovary Syndrome
PNX	Phoenixin-14
SD	Standard Deviation
SEM	Structural Equation Modeling
STROBE	Strengthening the Reporting of Observational Studies in Epidemiology
T2DM	Type 2 Diabetes Mellitus
WHO	World Health Organization
